# Supplementary material for: Molecular Impacts of Dietary Exposure to Nanoplastics Combined or Not with Arsenic in the Caribbean Mangrove Oysters (Isognomon alatus)
Source: Nanomaterials (Basel). 2021 Apr 28;11(5):1151. doi: 10.3390/nano11051151 (PMC8146365; doi:10.3390/nano11051151)
Supplement: Supplementary file 1 [file nanomaterials-11-01151-s001.zip › nanomaterials-1170542-SI.pdf]

**Supplementary Material**

# Molecular Impacts of Dietary Exposure to Nanoplastics Combined or Not with Arsenic in the Caribbean Mangrove Oysters (Isognomon Alatus)

**Marc Lebordais [1,2], Zélie Venel [1], Julien Gigault [3], Valerie S. Langlois [2] and Magalie Baudrimont [1,*]**

[1] Université de Bordeaux, CNRS, UMR EPOC 5805, Place du Dr Peyneau, 33120 Arcachon, France; zelie.venel@gmail.com (Z.V.); magalie.baudrimont@u-bordeaux.fr (M.B.)

[2] Centre Eau Terre Environnement, Institut national de la recherche scientifique (INRS), 490 rue de la Couronne Québec City, QC G1K 9A9, Canada; Marc.Lebordais@ete.inrs.ca (M.L.); Valerie.Langlois@inrs.ca (V.L.)

[3] Université Laval, UMI Takuvik 3376, 1045 avenue de la Médecine, Québec City, QC G1V 0A6, Canada; julien.gigault@takuvik.ulaval.ca

* Correspondence: magalie.baudrimont@u-bordeaux.fr; Tel.:+33-(0)-5-5622-3927

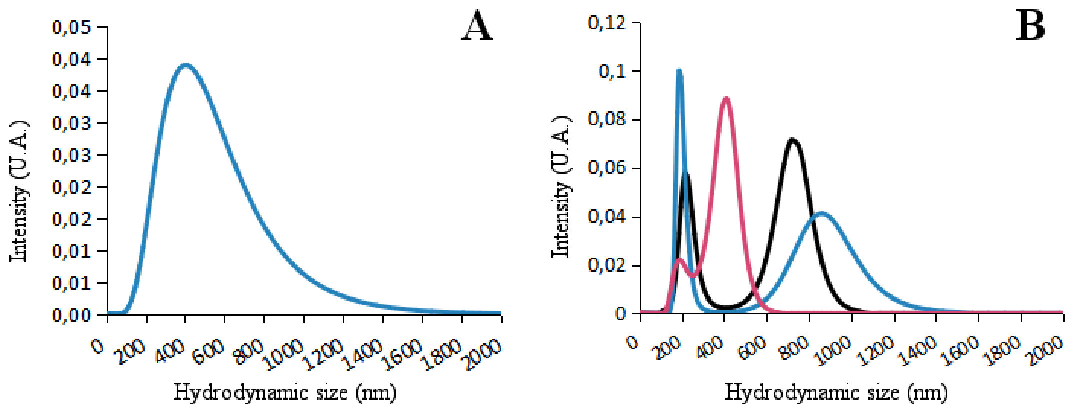


**Figure S1.** Size distribution of NPG nanoparticles measured by DLS on a Vasco Flex (using Sparse Bayesian Learning algorithm) showing the hydrodynamic size for overall nanoparticles in the dispersion (A), and the size distribution for each NPG batch presented with a different colour (B).

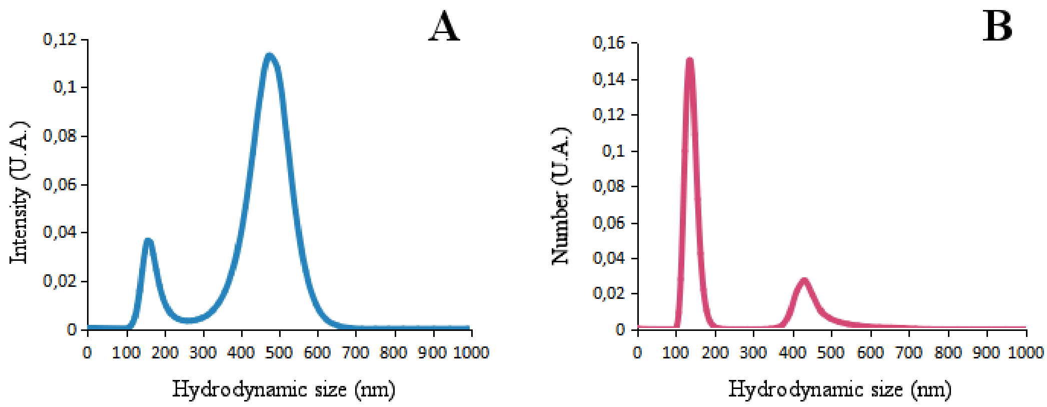


**Figure S2.** Size distribution of PSC nanoparticles measured by DLS on a Vasco Flex (using Sparse Bayesian Learning algorithm) showing the hydrodynamic size for overall nanoparticles in the dispersion (A), and the size distribution for each PSC batch (B).

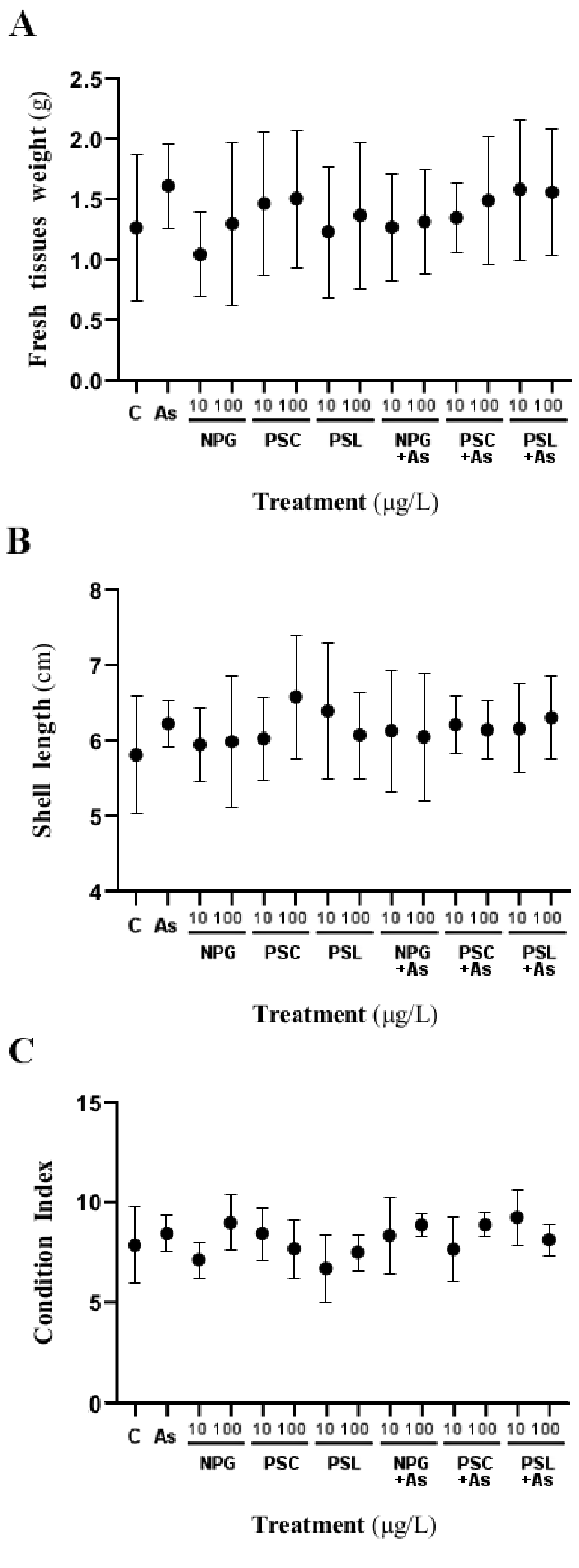


**Figure S3.** Comparisons of *I. alatus* biometric parameters after one week exposure (n = 4 ± sd). Fresh tissues weight in g (A), shell length in cm (B), and Condition Index (C).

**Table S1.** Primers used for quantifying gene expression by qPCR.

| Genes of interest | Full gene names<br>Forward (F) Reverse (R) primers | Associated cell functions |
|---|---|---|
| *β-actine** | Actin beta<br>F : AACGAGCGATTCAGATGTCC<br>R : CGATTCCTGGGTACATGGTT | Microfilaments |
| *rpl7** | Ribosomal protein L7<br>F : CCCAGGAAGGTCATGCAGTT<br>R : TCCCAGAGCCTTCTCGATGA | Ribosomal sub-unit |
| *cav* | Caveolin<br>F : CGTCGAGATCCAGACCTGTT<br>R : ACAGCATTGACTGCGTATGG | Endocytosis and vesicle transport |
| *cltc* | Clathrin heavy chain<br>F : AGACTCAGGACCCAGAGGAC<br>R : ATCACACGGGTTCTATCGGC | |
| *bax* | bcl2 associated X apoptosis regulator<br>F : AACTGGGGCAGAGTTGGATG<br>R : AATTGCTTCCCAGCCTCCTC | Cell cycle regulation |
| *gadd45* | Growth arrest DNA damage<br>F : TTGGCTTGACAAAAGTGCCG<br>R : CTGACAACCTGCATCTCGGT | |
| *p53* | Tumor protein p53<br>F : CGATGATCGGGTTCAGCAGA<br>R : GAGCTCTCTCAACACAGCCA | |
| *cat* | Catalase<br>F : CGAGGCTAGCCCAGACAAAA<br>R : TTGGGGAAATAGTTGGGGGC | Oxidative stress |
| *gapdh* | Glyceraldehyde-3-phosphate dehydrogenase<br>F : CACGGCAACACAGAAGGTTG<br>R : CCCTTCTGAAGTCGGCAAGT | |
| *sod1* | Superoxide dismutase Cu/Zn<br>F : AGACTGCGTCACATGCTTCA<br>R : GCGTCATGTAGGGGATCTGG | |
| *cox1* | Mitochondrial encoded cytochrome c oxidase 1<br>F : GTTGCCTTGGTCGCTAGACT<br>R : GAGCGTCTTGGGCTTAGTCA | Mitochondrial metabolism |
| *12S* | Mitochondrial encoded 12S rRNA<br>F : TCAGGTGTTACACAGCCGTC<br>R : GCAGGCGTTTTAATCCCGTC | |
| *mdr* | ATP Binding Cassette Subfamily<br>F : GCATGTTGCAAGCCTGTCAA<br>R : CAGTCAACTCAAGCAACCGC | Detoxification |

* : reference genes
